# Supplementary material for: Emerging unprecedented lake ice loss in climate change projections
Source: Nat Commun. 2022 Oct 2;13:5798. doi: 10.1038/s41467-022-33495-3 (PMC9527235; doi:10.1038/s41467-022-33495-3)
Supplement: Supplementary file 1 — Supplementary Information [file 41467_2022_33495_MOESM1_ESM.pdf]

Supplementary information

for

Emerging unprecedented lake ice loss in climate change projections

Lei Huang[1,2*], Axel Timmermann[1,2], Sun-Seon Lee[1,2], Keith B. Rodgers[1,2], Ryohei Yamaguchi[1,2], Eui-Seok Chung[3]

[1] Center for Climate Physics, Institute for Basic Science, Busan, South Korea

[2] Pusan National University, Busan, South Korea

[3] Korea Polar Research Institute, Incheon, South Korea

[*]Corresponding author: huanglei@pusan.ac.kr

This supplementary information includes Supplementary Note 1 and Figure S1-S9.

Supplementary Note 1

The relationship between air temperature and ice duration

As shown in Figure S2, there is a strong linear correlation (r value = -0.86) between freezing day (number of days when daily mean air temperatures are below 0°C) and annual mean air temperature over the lake regions in CESM2-LE. When the annual mean air temperature increases by 1°C on average, the freezing days decrease by 7.5 days. In addition, there is a strong linear correlation (r value = 0.89) between freezing day and ice duration. When the freezing day decreases by 1 day, the ice duration decreases by 0.8 day. Surface air warming clearly leads to the decrease of freezing days, thus leading to the shortening of the ice duration.

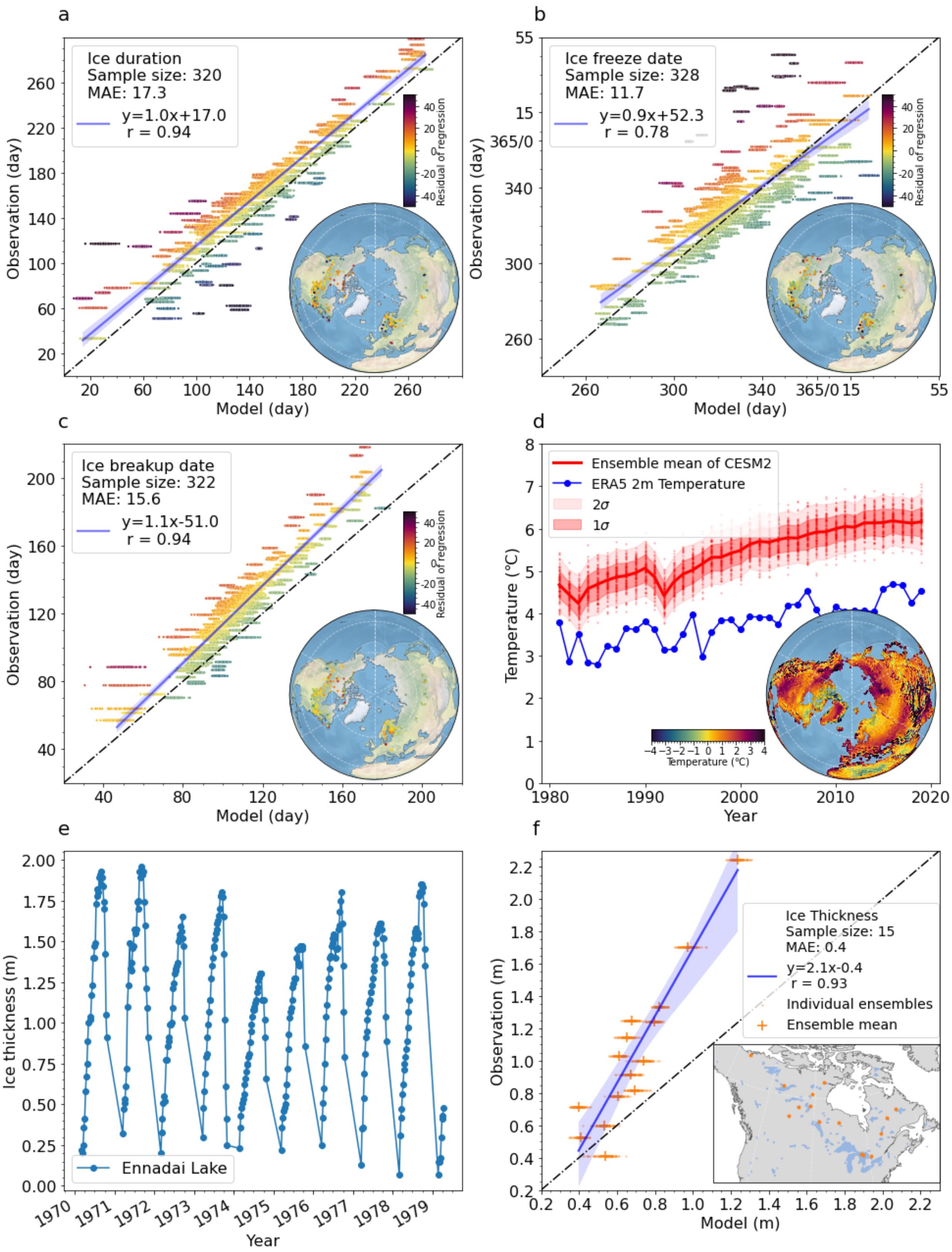

Figure S1. *Scatter plot of climatological mean of ice duration (a), freeze date (b) and breakup date (c). The x-axis is the model, and the y-axis is the observations. Results of all ensembles are*

plotted. The inset map shows the location of lakes (Supplementary Table S1), and color shading represents the regression residual. MAE is the abbreviation for mean absolute error. (d), Time series of the annual mean land surface air temperature in the 30-70°N area. Red is the model, and blue is the reanalysis product. The inset shows the climatological difference (1981-2020) between the model and the ERA5 reanalysis dataset. (e), The observational ice thickness in Ennadai Lake collected in the Canadian Ice Thickness Program. (f), Scatter plot of climatological mean of annually maximum ice thickness in observation (y-axis) and simulation (x-axis). The dots denote individual ensembles, and the crosses denote the ensemble mean. The inset map shows the location of lakes (Supplementary Table S2). Please note all correlation analysis in this figure is based on the ensemble mean of the simulation.

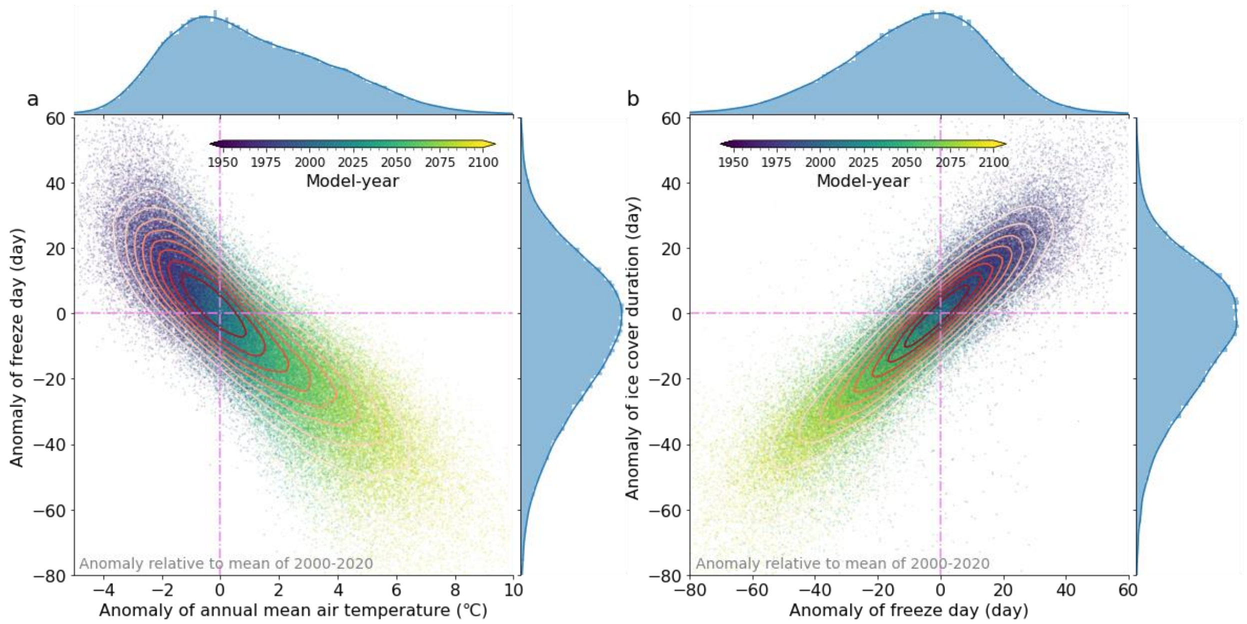

Figure S2. *(a), Same scatter plot as Figure 2a, except that the y-axis is replaced by the anomaly of freezing day (number of days when daily mean air temperatures are below 0°C). (b), Same scatter plot as (a), except that the x-axis is anomaly of freezing day, and the y-axis is the anomaly of ice duration.*

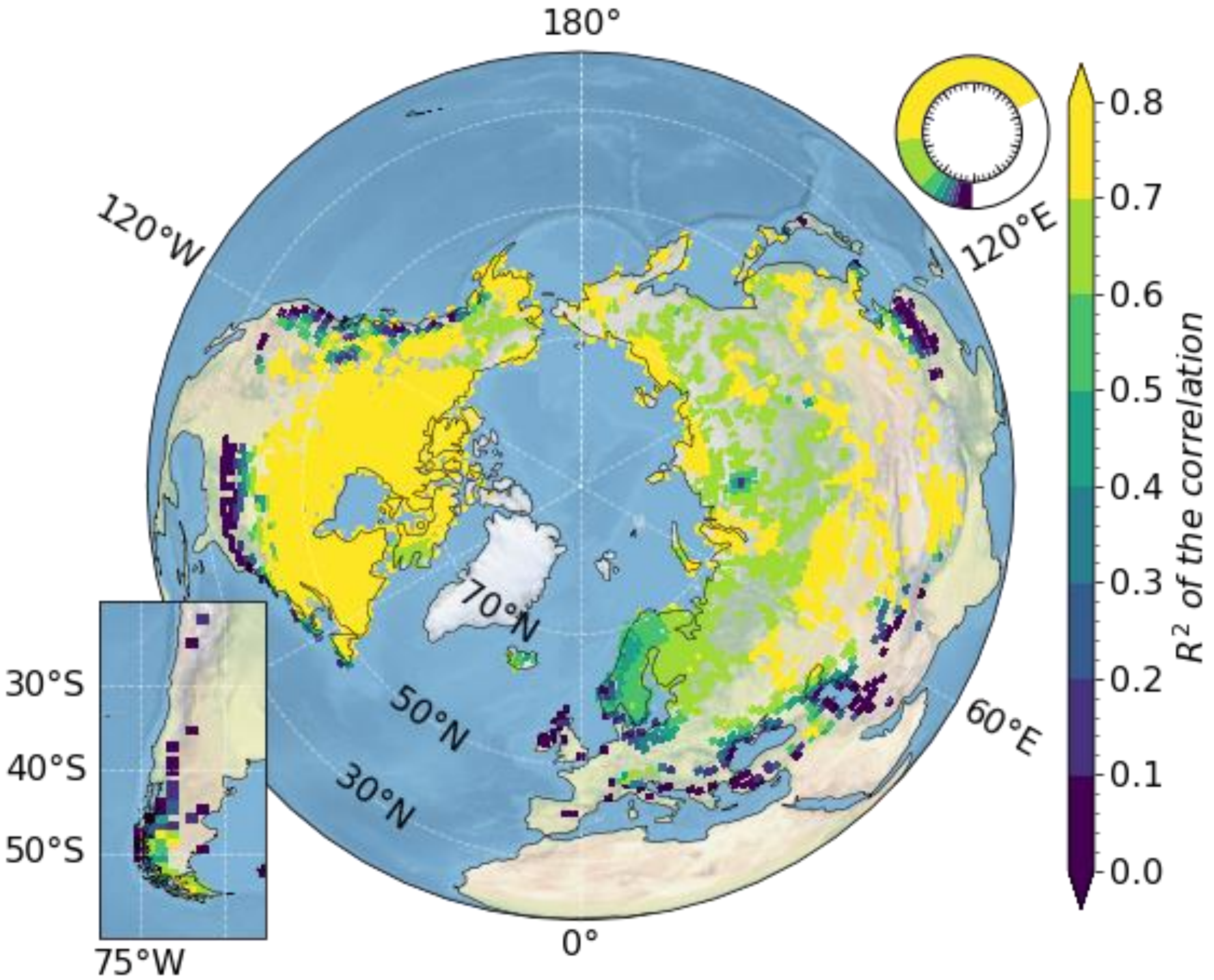

Figure S3. *R-square of the linear correlation between annual mean surface air temperature and ice duration over 2000-2100 in individual simulation grids, which implies the fraction of variance in ice duration explained the surface air temperature changes. The correlation analysis was performed on the aggregation of all ensemble members. In the intermittently frozen lakes, ice cover disappears in the middle of 2000-2100, thus continuous warming after permanent ice loss does not induce ice changes anymore. As a result, R-square in the intermittently ice-covered regions is low.*

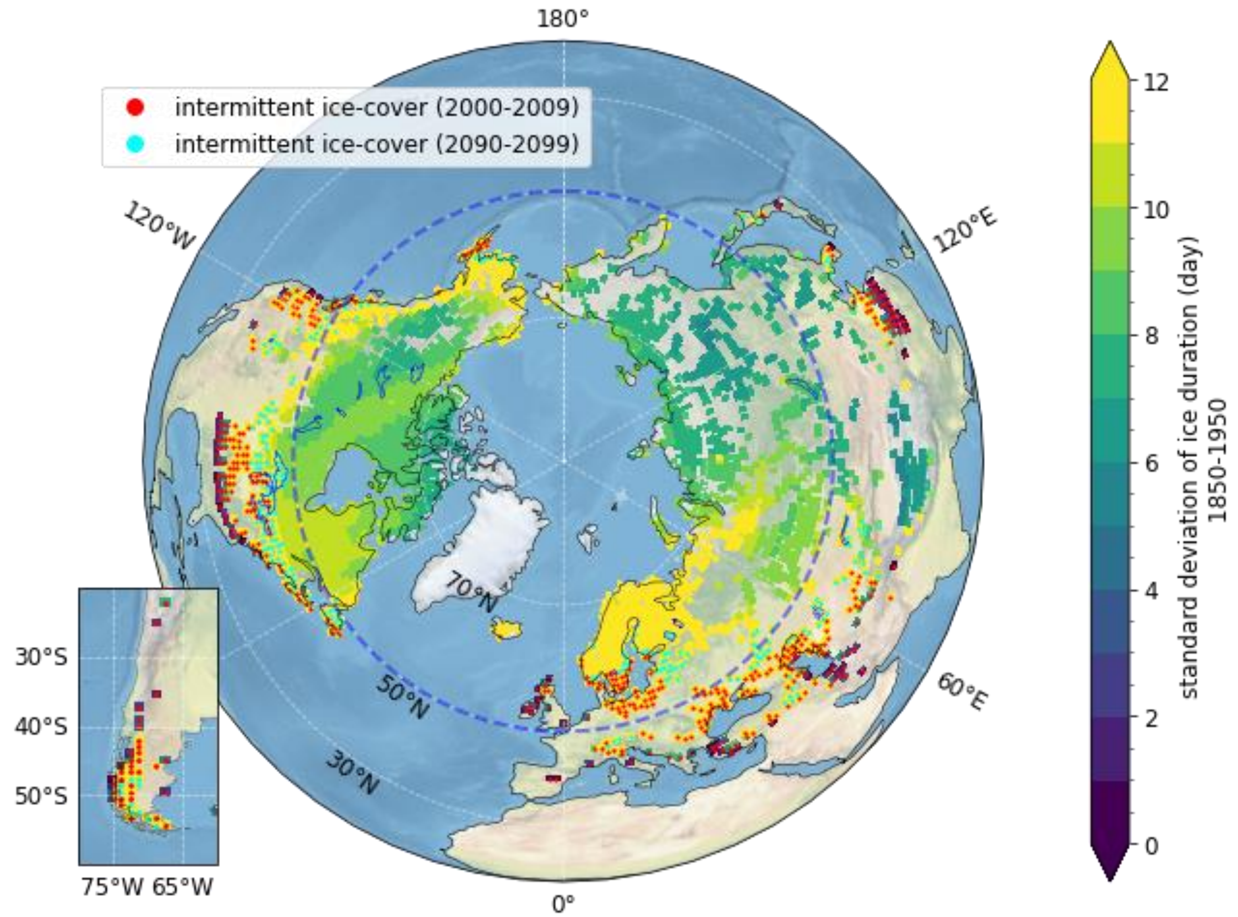

Figure S4. *Standard deviation of ice duration during 1850-1950 in the model results.*

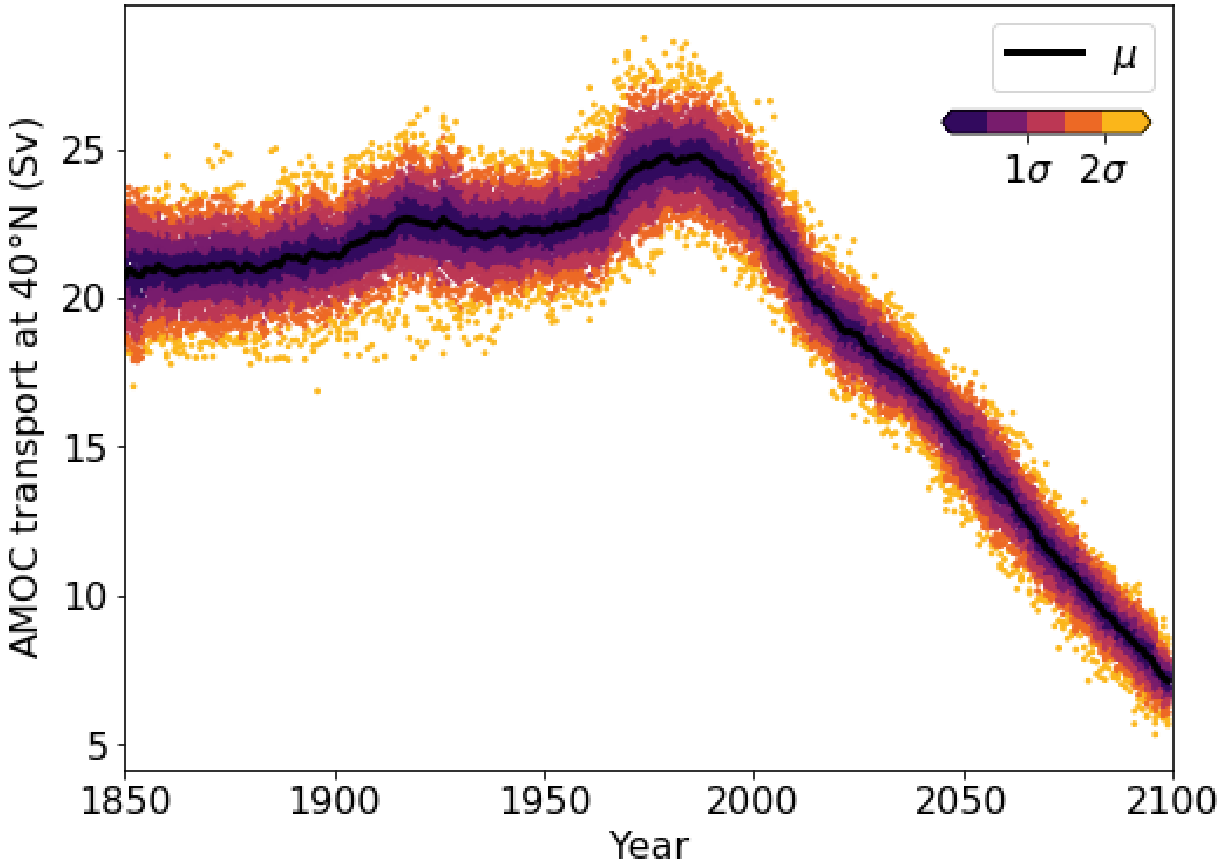

Figure S5. *Atlantic Meridional Overturning Circulation (AMOC) transport at 40°N.*

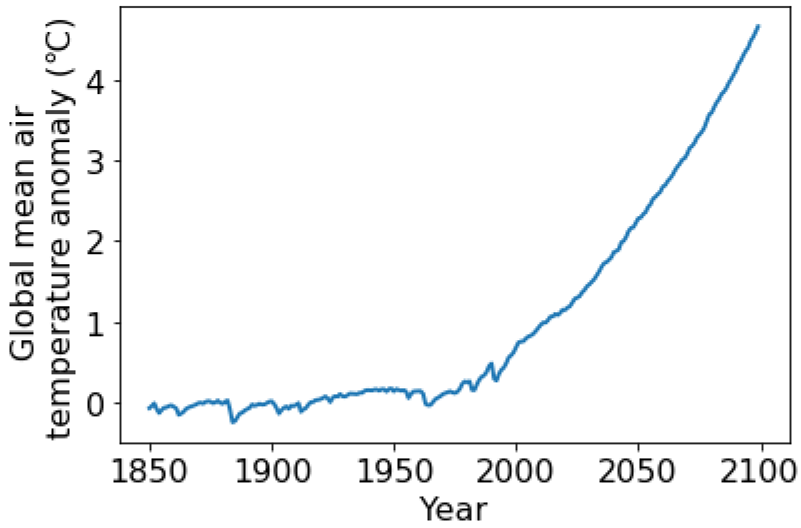

Figure S6. *Global mean surface air temperature anomalies relative to the climatological mean of 1850-1950.*

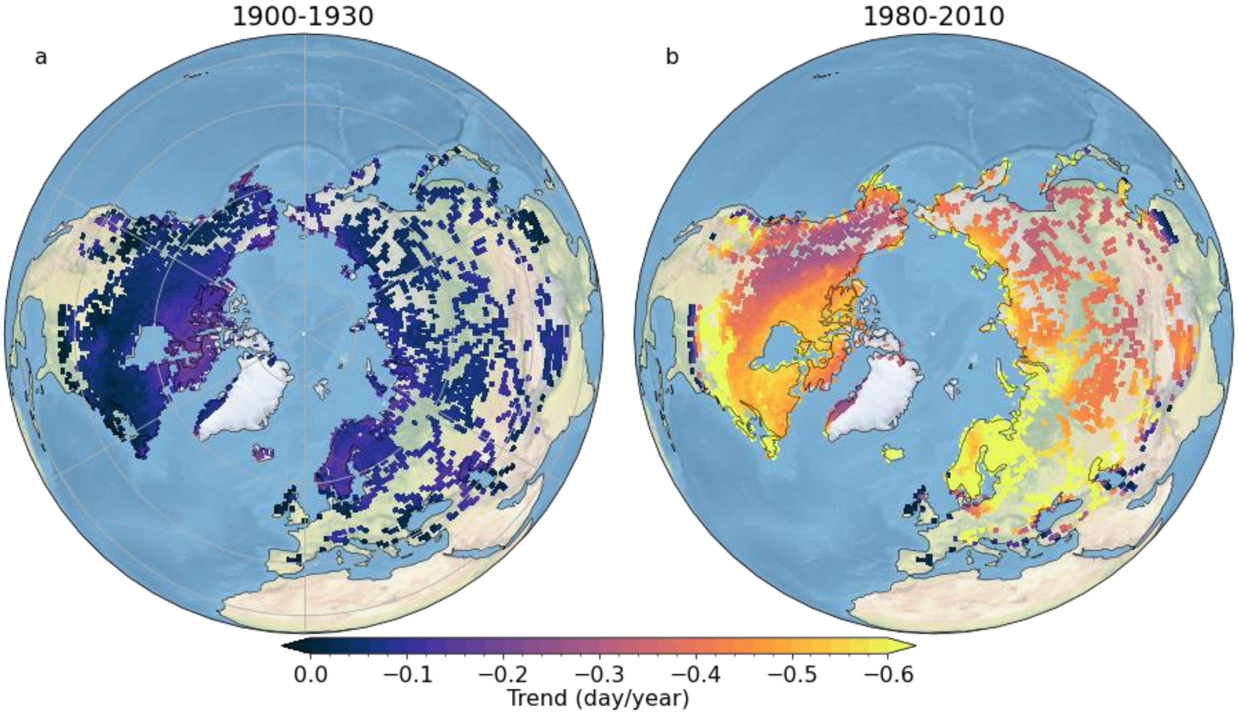

Figure S7. *The temporal trends of the ensemble mean for ice duration during 1900-1930 (a) and 1980-2010 (b), respectively.*

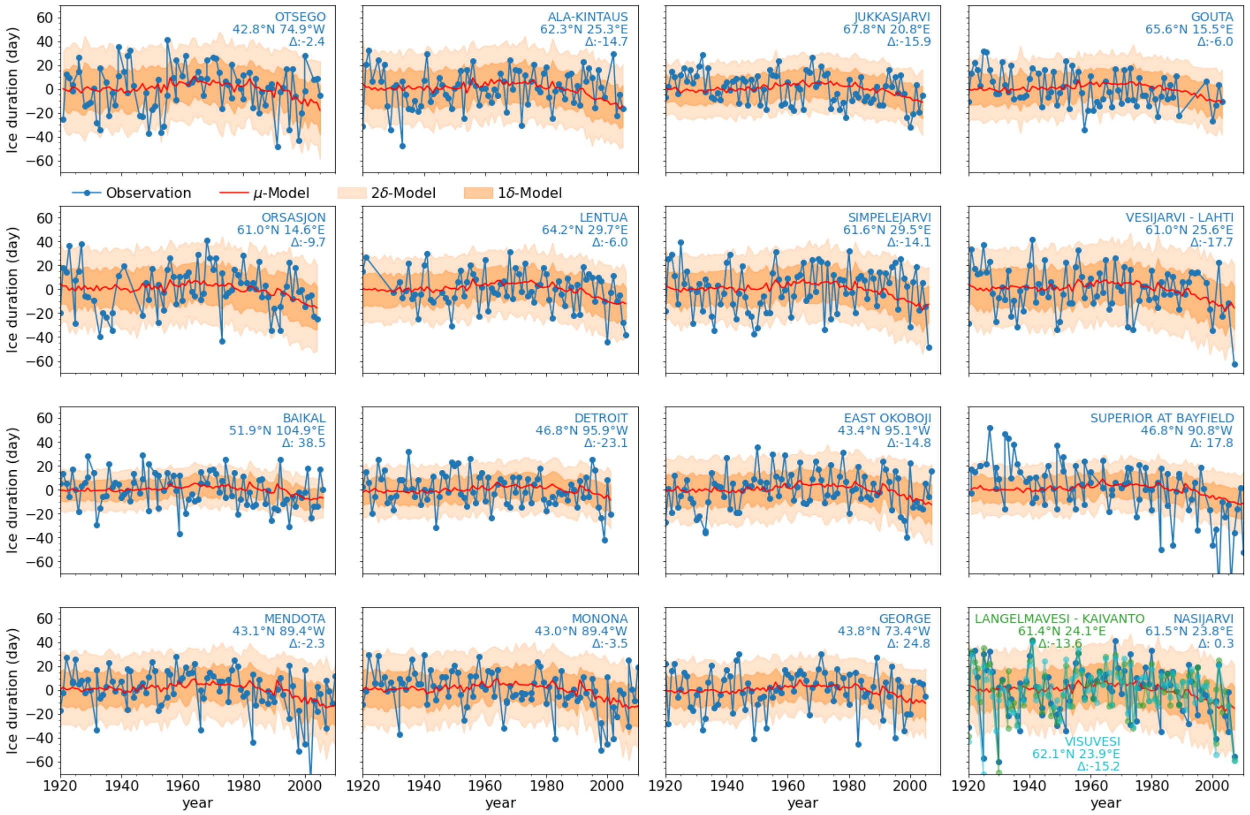

Figure S8. Time series of ice duration anomaly of individual lakes in the observation and simulation. The y-axis is the ice duration anomaly relative to climatological mean. The blue dots denote the observation. The red line denotes ensemble mean in the simulation, and the yellow shadings denote one and two standard deviations of all ensemble members around the ensemble mean. Δ denotes the difference of climatological mean between simulation and observation, while negative value indicates that our simulation underestimated ice duration. In the last panel, the observational records of three lakes in the same simulation grid are displayed.

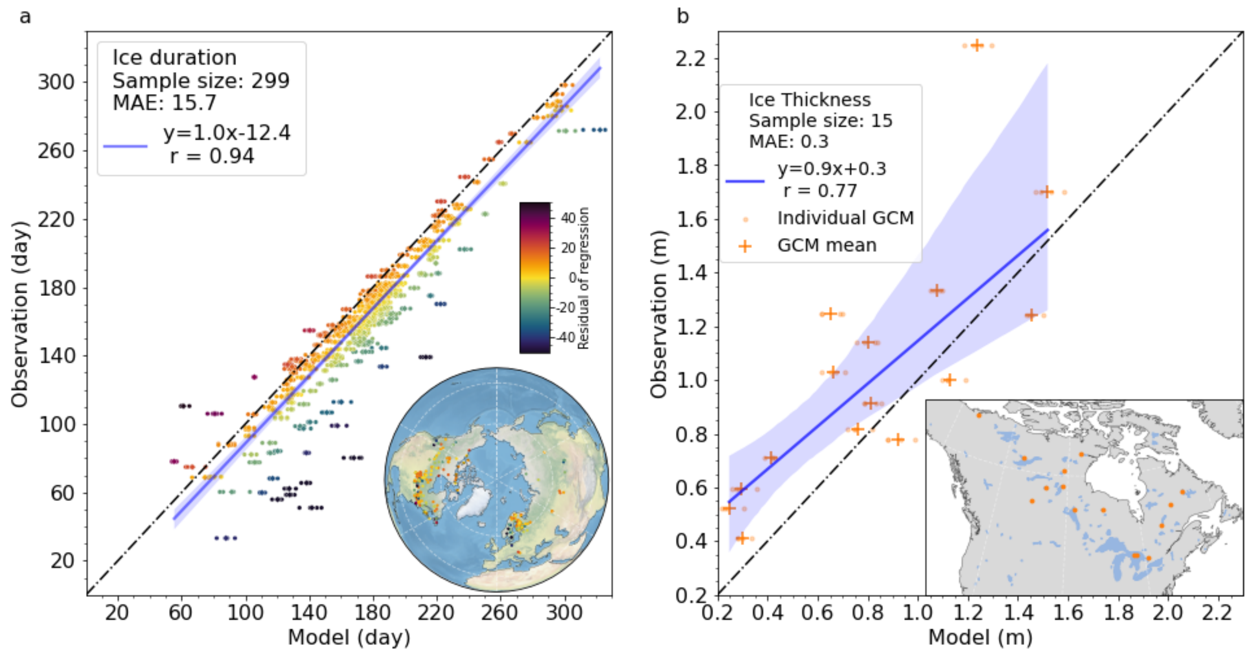

Figure S9. *Evaluation of climatological mean ice duration (a) and annually maximum ice thickness (b) for the SimStrat-UoG model in the ISIMIP 2b project. The x-axis is the model, and the y-axis is the observations. Results of simulations forced by four GCM output fields are all presented in (a) and (b). The correlation analysis was performed on the mean of four simulations.*
