# Supplementary material for: Emerging unprecedented lake ice loss in climate change projections
Source: Nat Commun. 2022 Oct 2;13:5798. doi: 10.1038/s41467-022-33495-3 (PMC9527235; doi:10.1038/s41467-022-33495-3)
Supplement: Supplementary file 3 — Description of Additional Supplementary Files [file 41467_2022_33495_MOESM3_ESM.pdf]

Description of Additional Supplementary Files

File Name: Supplementary Data 1
Description: Information of the observed lake ice phenology records used for the validation, e.g., lake name, geographic coordinates etc.

File Name: Supplementary Data 2
Description: Information of the measured ice thickness records used for the validation, e.g., lake name, geographic coordinates etc.
